# Short-term results of a phase II study of preoperative docetaxel/cisplatin/S-1 therapy for locally advanced gastric cancer

**DOI:** 10.1093/jjco/hyaa221

**Published:** 2020-12-03

**Authors:** Kazuhito Tsuchida, Tsutomu Sato, Toru Aoyama, Yosuke Atsumi, Kazuki Kano, Yukio Maezawa, Keisuke Kazama, Masakatsu Numata, Takanobu Yamada, Hiroshi Tamagawa, Hitoshi Murakami, Takashi Oshima, Hiroyuki Saeki, Haruhiko Cho, Norio Yukawa, Yuji Yamamoto, Munetaka Masuda, Yasushi Rino

**Affiliations:** Department of Surgery, Yokohama City University, 3-9, Fukuura, Kanazawa-ku, Yokohama City, Kanagawa, 236-0004 Japan; Department of Surgery, Tokyo Metropolitan Cancer and Infectious Diseases Center Komagome Hospital, 3-18-22, Honkomagome, Bunkyo-ku, Tokyo, 113-8677 Japan; Department of Surgery, Yokohama City University, 3-9, Fukuura, Kanazawa-ku, Yokohama City, Kanagawa, 236-0004 Japan; Department of Surgery, Yokohama City University, 3-9, Fukuura, Kanazawa-ku, Yokohama City, Kanagawa, 236-0004 Japan; Department of Surgery, Yokohama City University, 3-9, Fukuura, Kanazawa-ku, Yokohama City, Kanagawa, 236-0004 Japan; Department of Surgery, Yokohama City University, 3-9, Fukuura, Kanazawa-ku, Yokohama City, Kanagawa, 236-0004 Japan; Department of Surgery, Yokohama City University, 3-9, Fukuura, Kanazawa-ku, Yokohama City, Kanagawa, 236-0004 Japan; Department of Surgery, Tokyo Metropolitan Cancer and Infectious Diseases Center Komagome Hospital, 3-18-22, Honkomagome, Bunkyo-ku, Tokyo, 113-8677 Japan; Department of Surgery, Yokohama City University, 3-9, Fukuura, Kanazawa-ku, Yokohama City, Kanagawa, 236-0004 Japan; Department of Surgery, Yokohama City University, 3-9, Fukuura, Kanazawa-ku, Yokohama City, Kanagawa, 236-0004 Japan; Department of Surgery, Yokohama City University, 3-9, Fukuura, Kanazawa-ku, Yokohama City, Kanagawa, 236-0004 Japan; Department of Surgery, Yokohama City University, 3-9, Fukuura, Kanazawa-ku, Yokohama City, Kanagawa, 236-0004 Japan; Department of Surgery, Yokohama City University, 3-9, Fukuura, Kanazawa-ku, Yokohama City, Kanagawa, 236-0004 Japan; Department of Surgery, Yokohama City University, 3-9, Fukuura, Kanazawa-ku, Yokohama City, Kanagawa, 236-0004 Japan; Department of Surgery, Yokohama City University, 3-9, Fukuura, Kanazawa-ku, Yokohama City, Kanagawa, 236-0004 Japan; Department of Surgery, Yokohama City University, 3-9, Fukuura, Kanazawa-ku, Yokohama City, Kanagawa, 236-0004 Japan; Department of Surgery, Tokyo Metropolitan Cancer and Infectious Diseases Center Komagome Hospital, 3-18-22, Honkomagome, Bunkyo-ku, Tokyo, 113-8677 Japan; Department of Surgery, Yokohama City University, 3-9, Fukuura, Kanazawa-ku, Yokohama City, Kanagawa, 236-0004 Japan; Department of Surgery, Yokohama City University, 3-9, Fukuura, Kanazawa-ku, Yokohama City, Kanagawa, 236-0004 Japan; Department of Surgery, Yokohama City University, 3-9, Fukuura, Kanazawa-ku, Yokohama City, Kanagawa, 236-0004 Japan; Department of Surgery, Yokohama City University, 3-9, Fukuura, Kanazawa-ku, Yokohama City, Kanagawa, 236-0004 Japan

**Keywords:** gastric cancer, preoperative chemotherapy, docetaxel/cisplatin/S-1, locally advanced cancer

## Abstract

**Background:**

A multi-institutional phase II study was conducted to evaluate the efficacy and safety of preoperative docetaxel, cisplatin and S-1 therapy in marginally resectable advanced gastric cancer.

**Methods:**

Patients with macroscopic type 4, large macroscopic type 3 and bulky lymph node metastasis received two cycles of preoperative docetaxel, cisplatin and S-1 therapy (docetaxel 40 mg/m^2^ and cisplatin 60 mg/m^2^ on day 1, and S-1 80 mg/m^2^ for 14 days, every 4 weeks). The primary endpoint was the pathological response rate, with an expected value of 65%.

**Results:**

Thirty-one patients were enrolled in this study. The pathological response rate was 54.8%, and it was higher than the threshold value but lower than the expected rate. The R0 resection rate was 93.5%. The frequencies of grade 3–4 toxicities during docetaxel, cisplatin and S-1 therapy were 41.9% for neutropenia, 6.5% for febrile neutropenia and 32.3% for nausea/vomiting. Grade 2 and 3 surgical morbidities occurred in 23.3 and 6.7% of the patients, respectively.

**Conclusions:**

Preoperative docetaxel, cisplatin and S-1 therapy was feasible in terms of chemotherapy-related toxicities and surgical morbidity, but the effect did not achieve the expected value. The association between the pathological response rate and survival will be evaluated in the final analysis of this clinical trial.

## Teaser

A phase II study of preoperative docetaxel/cisplatin/S-1 therapy for locally advanced gastric cancer was feasible, but the pathological response rate did not achieve the expected value.

## Introduction

Gastric cancer is the fifth most common cancer and the third leading cause of cancer-related mortality. An estimated 1033 700 new gastric cancer cases and 782 700 deaths occurred (1). Curative resection with perioperative adjuvant treatment is the standard treatment for locally advanced gastric cancer worldwide (2). In Asia, D2 gastrectomy and postoperative adjuvant chemotherapy with S-1, S-1 and docetaxel, or capecitabine and oxaliplatin is the standard treatment (3). However, the recurrence rate was reported to 30–40%, even after curative treatment. In addition, it was previously reported that macroscopic type 4, large (diameter >8 cm) macroscopic type 3, and bulky lymph node metastasis have been reported as prognostic factors in locally advanced gastric cancer (4,5). Aggressive treatment is needed to improve the survival of patients with locally advanced gastric cancer who have risk factors for severe disease.

Curative resection and perioperative chemotherapy are widely accepted treatments in Europe (6). Theoretically, preoperative (neoadjuvant) chemotherapy has some clinical benefits in comparison to postoperative chemotherapy (7). Preoperative chemotherapy might be able to eliminate micrometastasis and improve chemotherapy compliance in comparison to postoperative chemotherapy. However, the optimal regimen and duration are unclear.

Among several promising regimens, combination treatment with docetaxel, cisplatin and S-1 (DCS) had received attention for its efficacy and safety in patients with metastatic gastric cancer (8,9) until the JCOG 1013 study denied the superiority of DCS therapy over cisplatin plus S-1 (CS) therapy in 2019 (10). Based on this background, we conducted a multi-institutional phase II trial of preoperative chemotherapy with DCS to evaluate the efficacy and safety in gastric cancer patients with type 4 tumors, large type 3 tumors or bulky lymph node metastasis.

The present study evaluated the efficacy of preoperative DCS therapy. The primary endpoint was the pathological response rate (pRR), and the secondary endpoints were chemotherapy-related toxicities, morbidity and mortality as short-term outcomes and the overall and relapse-free survival as long-term outcomes. This report clarified the impact of DCS on short-term endpoints.

## Patients and methods

### Eligibility criteria

Tumors were staged according to the 13th edition of the Japanese Gastric Cancer Classification (11). However, in this paper, we translated the stages to the 14th edition (12), which was integrated with the 7th edition of AJCC cancer staging (13). The eligibility criteria were as follows: (i) histologically proven gastric adenocarcinoma, (ii) macroscopic type 4, large (major axis >8 cm) macroscopic type 3, or bulky lymph node metastasis (one lymph node **≥**3 cm or two adjacent lymph nodes **≥**1.5 cm along the celiac, common or proper hepatic, or splenic arteries), (iii) macroscopically resectable gastric cancer without distant metastasis diagnosed by thoracic/abdominal/pelvic computed tomography and staging laparoscopy, (iv) esophageal invasion <3 cm, (v) age 20–80 years, (vi) Eastern Cooperative Oncology Group performance status 0–1, (vii) no previous history of chemotherapy or radiation therapy for any other cancer, and no previous history of surgery for gastric cancer, with the exception of bypass surgery, (viii) an adequate organ function (white blood cell [WBC] count ≥3000/mm^3^ and ≤12 000/mm^3^; hemoglobin level ≥9.0 g/dl; platelet count ≥100 000/mm^3^; aspartate aminotransferase [AST] and alanine aminotransferase [ALT] levels ≤2.5x the upper limit of normal [ULN]; total bilirubin level ≤1.5 mg/dL; creatinine ≤1.5 mg/dL and creatinine clearance ≥60 ml/min/body in the Cockcroft–Gault equation) and (ix) written informed consent.

The exclusion criteria were as follows: (i) synchronous or metachronous cancer within 5 years, (ii) severe co-morbidities, (iii) pregnant or lactating woman, (iv) severe mental disease that might hinder participation in this study or (v) physician judged that the patient would be unable to complete the protocol treatment.

### Preoperative chemotherapy

We adopted the DCS regimen reported by Koizumi et al. (9) because of its low toxicity and high response rate among various DCS regimens. Docetaxel (40 mg/m^2^) and cisplatin (60 mg/m^2^) were administered by intravenous infusion on day 1 of each cycle, and S-1 (80 mg/m^2^) was given orally for the first 14 days of a 28-day cycle. After the completion of two cycles of chemotherapy or when the continuation of chemotherapy was difficult, the possibility of curative resection was evaluated. If macroscopically curative resection was considered possible, the patients underwent surgery within 42 days after the last administration of S-1.

Chemotherapy-related toxicities were evaluated according to the Common Terminology Criteria for Adverse Events, version 3.0. The subsequent chemotherapy cycle was delayed until patient recovery, including the following parameters: WBC count ≥3000/mm^3^; neutrophil count ≥1500/mm^3^; platelet count ≥100 000/mm^3^; AST and ALT levels ≤2.5x ULN; total bilirubin level ≤1.5x ULN; creatinine level ≤1.5 mg/dL and creatinine clearance ≥60 ml/min/body; diarrhea or stomatitis grade ≤1; and other non-hematological adverse event grade ≤2. The DCS doses were reduced in the event of a WBC count ≤1000/mm^3^; neutrophil count ≤500/mm^3^; platelet count ≤50 000/mm^3^; or grade ≥3 non-hematological adverse events.

### Surgery and adjuvant chemotherapy

After laparotomy, intraperitoneal lavage cytology was examined. Regardless of the results of cytology, gastrectomy with D2 lymph node dissection was performed if macroscopic curative resection was possible without obvious residual cancer. Laparoscopic gastrectomy was not allowed, and combined resection of the surrounding organs was allowed if R0 resection was considered possible. If curative resection was considered impossible due to factors other than positive lavage cytology, the protocol treatment was terminated. Surgical morbidities were evaluated by the Clavien–Dindo classification (14) in this analysis.

Adjuvant chemotherapy with S-1 was started within 42 days after surgery for patients who underwent R0 resection or R1 resection with positive lavage cytology. The oral administration of S-1 (80 mg/m^2^) for 28 days followed by 14 days rest was continued for 1 year after surgery.

**Figure 1.**
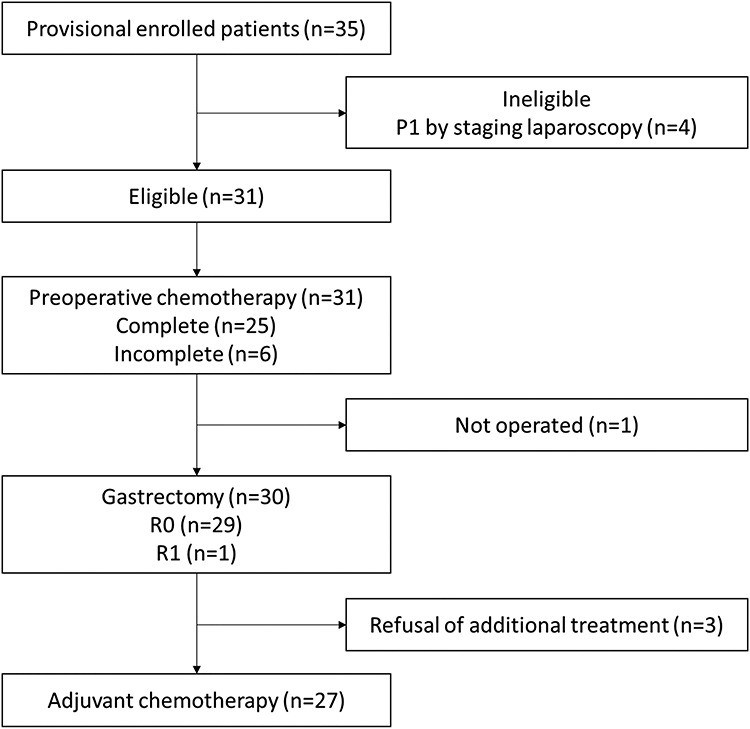
Flow diagram of the present study.

### Study design and statistical methods

This study was designed to evaluate the efficacy and safety of preoperative DCS therapy in patients with locally advanced gastric cancer. The primary endpoint was the pRR, and the secondary endpoints were chemotherapy-related toxicities, morbidity and mortality as short-term outcomes and the overall and relapse-free survival as long-term outcomes. Because the required follow-up period has not yet been reached, long-term results are premature and will be reported by 2022.

The pathological response of resected specimens was graded by a histopathologist according to the 13th edition of the Japanese classification of gastric carcinoma as follows: grade 0, no evidence of effect; grade 1a, viable tumor cells occupy ≥2/3 of the tumorous area; grade 1b, viable tumor cells remaining in ≥1/3 but <2/3; grade 2, viable tumor cells remaining in <1/3; grade 3, no viable tumor cells remaining (11). All cases were diagnosed by a pathologist at each institution who had a specialist qualification from the Japanese Society of Pathology. A grade 1b-3 response was defined as a positive pathological response in this study. The clinical response was evaluated by the Response Evaluation Criteria in Solid Tumor version 1.0 (15), and the disease control rate was defined as the sum of the complete response, partial response and stable disease rates.

The pRR of preoperative chemotherapy with CS for type 4 tumors or large type 3 tumors in JCOG 0210 and for bulky or paraaortic lymph node metastasis in JCOG 0405 were 49 and 51%, respectively (16,17). DCS therapy was expected to be more effective but more toxic than CS therapy. Based on these data, we calculated that the required number of patients was 25 patients, assuming that the threshold and expected pRR were 40 and 65%, respectively, with a one-sided α error of 0.05 and a β error of 0.2. Considering the possibility of dropouts and ineligible patients, 30 patients were required.

The protocol of this study was approved by the protocol review committee of Yokohama City University (approved number: B09003120025) and each participating institution, in accordance with the ethical standards prescribed by the Helsinki Declaration. This study was registered at the University Hospital Medical Information Network (ID: UMIN000003052).

## Results

### Patients

Between August 2008 and May 2016, 31 patients from three institutions were enrolled in this study. Figure 1 shows the flow diagram of this study, and Table 1 shows the patient and tumor characteristics. All patients underwent staging laparoscopy before enrollment, with 29 patients having P0CY0 and 2 having P0CY1. The rate of completion of preoperative chemotherapy was 80.6% (25 of 31). Preoperative chemotherapy was discontinued in five patients (16.1%) due to adverse events. Thirty patients received D2 gastrectomy. One patient did not proceed to surgery due to progression of metastatic lymph node disease during preoperative chemotherapy. The disease control rate was 96.8% (30 of 31).

**Table 1:** Patient and tumor characteristics according to the 14th edition of Japanese classification of gastric carcinoma

Characteristic	Variable	Number of patients (*n* = 31)	(%)
Age (years)	Median	69	
	Range	52–78	
Gender	Male/Female	23/8	74.2/25.8
PS	0/1	28/3	90.3/9.7
Macroscopic type	Type 3	25	80.6
	Type 4	5	16.1
	Others	1	3.2
Histological type	Differentiated	12	38.7
	Undifferentiated	19	61.3
Clinical T factor	T2	1	3.2
	T3	4	12.9
	T4a	25	80.6
	T4b	1	0.3
Clinical N factor	N0	2	6.5
	N1	13	41.9
	N2	16	51.6
	N3	0	0.0
CY factor	CY0	29	93.5
	CY1	2	6.5
Clinical M factor	M0	29	93.5
	M1	2	6.5
Clinical Stage	Stage IA	0	0.0
	Stage IB	1	3.2
	Stage IIA	0	0.0
	Stage IIB	1	3.2
	Stage IIIA	15	48.3
	Stage IIIB	12	38.7
	Stage IIIC	0	0.0
	Stage IV	2	6.5

PS: performance status; CY: peritoneal lavage cytology

### Response

Table 2 shows the pRR of the primary tumors. A pathological response, defined as a complete response or <2/3 residual cancer cells (grade 1b-3), was 54.8% (17 of 31). The pathological complete response rate (grade 3) was 3.2% (1 of 31).

**Table 2:** Pathological response of the primary tumors

Characteristics	Number of patients (*n* = 31)	(%)
Grade 0	2	6.4
Grade 1a	11	35.5
Grade 1b	10	32.3
Grade 2	6	19.4
Grade 3	1	3.2
Unresected	1	3.2

### Chemotherapy-related Toxicities

Table 3 shows the details of preoperative chemotherapy-related toxicities. The incidence of grade 3 or 4 toxicities was high for leukopenia, neutropenia, and nausea/vomiting. However, the incidence of febrile neutropenia (FN) was relatively low and there were no chemotherapy-related deaths. The actual total dose of each drug that was administered is shown in Table 4.

### Surgery

Table 5 shows the details of the surgical procedures that were performed. The proximal margin was positive in one patient, but no additional treatment was given because of the patient’s refusal. D2 lymph node dissection was performed in all patients, but there were no patients who underwent extended lymph node dissection. Intraperitoneal lavage cytology was negative in all patients, including two patients in whom lavage cytology changed from positive to negative after preoperative chemotherapy. The R0 resection rate was 93.5% (29 of 31). Grade 2 surgical morbidities were observed in five (16.7%) patients, and grade 3a surgical morbidities were observed in two (6.7%) patients. No patients required reoperation for morbidity or surgical mortality. The details of the postoperative complications are shown in Table 6, and the details of the pathological findings are shown in Table 7.

**Table 3:** Adverse events related to preoperative chemotherapy

Adverse event	Grade 3/4		All grade	
	Number of patients	(%)	Number of patients	(%)
Hematological				
Leukopenia	7	22.5	20	64.5
Neutropenia	13	41.9	23	74.2
Anemia	4	12.9	21	67.7
Thrombocytopenia	1	3.2	3	9.7
Febrile neutropenia	2	6.5	2	6.5
No hematological				
Elevated AST	0	0.0	7	22.6
Elevated ALT	0	0.0	9	29.0
Elevated serum Bil	0	0.0	1	3.2
Elevated creatine	0	0.0	11	35.5
Nausea/Vomiting	10	32.3	29	93.5
Diarrhea	3	9.7	14	45.2
Rash	0	0.0	0	0.0
Fatigue	3	9.7	26	83.9
Alopecia	0	0.0	26	83.9
Thromboembolism	1	3.2	1	3.2
Infusion related reaction	0	0.0	0	0.0
Paresthesia	0	0.0	0	0.0

AST: Aspartate transaminases; ALT: Alanine aminotransferase; Bil: Bilirubin.

**Table 4:** Actual total dose administered

Treatment parameter	S-1	Cisplatin	Docetaxel
Mean	82.5%	86.5%	88.0%
≥90%	19	21	21
≥80 to <90%	3	2	3
≥60 to <80%	3	2	0
<60%	6	6	7

**Table 5:** Surgical outcomes

Characteristics	Number of patients (*n* = 30)	(%)
Type of gastrectomy		
Total	25	83.3
Distal	5	16.7
R category		
R0	29	96.7
R1	1	3.3
R2	0	0.0
Lymph node dissection		
D1+	0	0.0
D2	30	100.0
Combined organ resection		
Spleen	14	46.7
Gallbladder	2	6.7
Others	4	13.3
Bleeding (g)		
Median	629	
Range	298–1352	
Operation time (min)		
Median	249	
Range	155–459	

**Table 6:** Postoperative complications

	All grades	(%)	Grade 3a	(%)
Postoperative bleeding	1	3.3	0	0.0
Anatomic leakage	1	3.3	0	0.0
Pancreatic fistula	1	3.3	1	3.3
Intra-abdominal abscess	1	3.3	1	3.3
Wound infection	0	0.0	0	0.0
Ileus	1	3.3	0	0.0
Anatomic stenosis	1	3.3	0	0.0
Pneumothorax	1	3.3	1	3.3
Pneumonia	2	6.7	0	0.0

**Table 7:** Pathological findings according to the 14th edition of Japanese classification of gastric carcinoma

Characteristic	Variable	Number of patients (*n* = 30)	(%)
Pathological T factor	ypT0	1	3.3
	ypT1	4	13.3
	ypT2	2	6.7
	ypT3	12	40.0
	ypT4a	11	36.7
	ypT4b	0	0.0
Pathological N factor	ypN0	10	33.3
	ypN1	1	3.3
	ypN2	9	30.0
	ypN3a	7	23.3
	ypN3b	3	10.0
Number of resected lymph nodes	Median	38	
	Range	22–72	
CY factor	CY0	30	100.0
	CY1	0	0.0
Pathological M factor	M0	30	100.0
	M1	0	0.0
Pathological stage	ypStage 0	1	3.3
	ypStage IA	3	10.0
	ypStage IB	1	3.3
	ypStage IIA	6	20.0
	ypStage IIB	2	6.7
	ypStage IIIA	7	23.3
	ypStage IIIB	4	13.3
	ypStage IIIC	6	20.0
	ypStage IV	0	0.0

CY: peritoneal lavage cytology

### Postoperative chemotherapy

Postoperative adjuvant chemotherapy with S-1 was administered to 27 of the 29 patients who underwent R0 resection. Two patients with R0 resection and 1 with R1 refused additional treatment (Figure 1). Of the 27 patients who received postoperative adjuvant chemotherapy, 13 (48.1%) completed postoperative chemotherapy with S-1 for 1 year, whereas 8 discontinued treatment due to toxicity, and 6 discontinued it due to recurrence.

## Discussion

The aim of the present study was to evaluate the efficacy and safety of preoperative chemotherapy with DCS in patients with marginally resectable advanced gastric cancer, including patients with type 4 tumors, larger type 3 tumors or bulky lymph node metastasis. Although the chemotherapy-related toxicities and surgical complications as secondary endpoints were feasible, the pRR as the primary endpoint was higher than the threshold value but lower than the expected rate. These results suggested that our DCS regimen was less toxic but not sufficiently effective to justify proceeding to a phase III trial.

The most likely reason why our DCS regimen failed to achieve the primary endpoint was that the total dose was insufficient. DCS regimens with higher and lower doses have been reported for unresectable advanced gastric cancer, with the higher-dose regimen being more effective but more toxic and the lower-dose regimen being less toxic (8,9). In Japan, several phase II trials have been conducted to identify the optimal dosage of DCS for preoperative chemotherapy (18–21). Each study had different eligibility criteria and primary endpoints. Among the studies of preoperative low-dose DCS therapy, we first set the pRR as the primary endpoint. This was not a common approach at the time, as we did not have sufficient evidence regarding the pathological response, but a recent study showed that the pRR was an excellent surrogate endpoint for the overall survival in studies of preoperative chemotherapy (22). The doses of each drug in this study were as follows: docetaxel, 10 mg/m^2^/week; cisplatin, 15 mg/m^2^/week and S-1, 280 mg/m^2^/week. On the other hand, a representative high-dose regimen reported by Hirakawa et al. (18) was composed of docetaxel 20 mg/m^2^/week, cisplatin 20 mg/m^2^/week and S-1 373 mg/m^2^/week. Recently, the pRR of other studies of preoperative DCS therapy was reported to be 50–57% in the lower-dose regimen (19,20) and 65.9% in the higher-dose regimen (18). The pRR in this study was 54.8%. Similar pRRs were observed in other studies with low-dose DCS regimens. We speculate that this supports the hypothesis that the pRR is associated with the dose of chemotherapy.

The feasibility of two-course chemotherapy was also controversial in terms of the total dose. In this study, patients received two courses of preoperative DCS therapy. In the KDOG 1001 study, which used the same doses as this study, the overall pRR was 57.5%, but it was higher (69.5%) in patients who received four courses (20). However, according to their protocol, it was possible to administer up to four courses if a good response was obtained. This may indicate that patients who had already shown a histological response by the second course received four courses of chemotherapy. Meanwhile, Aoyama et al. (21) reported that the pRR of four courses of DCS therapy did not differ from that of two courses, according to the results of a phase II trial comparing neoadjuvant chemotherapy with two and four courses for locally advanced gastric cancer. Considering these reports, more careful discussion is needed to increase the number of courses of preoperative chemotherapy.

In this study, chemotherapy-related toxicities were relatively mild. In particular, the incidence of FN was 6.5%, which was similar to the results of the JCOG 1002 (5.7%) and KDOG 1001 (7.5%) trials, which used the same dosing regimen as this study (19,20). Conversely, the incidence of FN in patients treated with higher-dose DCS therapy was reported to be 16.3% (18). Generally, FN requires a long time to recover, and the long chemotherapy-free interval may help tumor progression and prevent subsequent surgery. Fortunately, gastrectomy was performed in 30 of 31 patients (96.8%) in the present study, with one patient unable to undergo gastrectomy due to tumor progression.

Postoperative complications have been reported to have a negative impact on the long-term survival (23). The frequency of postoperative complications of **≥** grade 3 has been reported to be 8.3–12% in previous studies of doublet preoperative chemotherapy (16,17). There was a concern that postoperative complications would increase because this study adopted a triplet regimen. However, the actual incidence of grade ≥2 and ≥3 postoperative complications in this study was 23.3 and 6.7%, respectively, and there was no surgical mortality. We hypothesize that in addition to the administration of a low-dose regimen, the volume reduction induced by preoperative chemotherapy contributed to the safety.

The present study was associated with some limitations. First, this was a single-arm phase II study and evaluated only early endpoints, including the pathological response, chemotherapy-related toxicities, and surgical results. Second, the pRR was determined by the pathologist at each institution and there was no central assessment. Third, the low incidence of postoperative complications may reflect the difficulty of the surgical procedure rather than the impact of the intensity of preoperative chemotherapy. In this study, none of the patients required extended lymph node dissection, even though patients with marginally resectable cancer were included.

In conclusion, preoperative DCS therapy was feasible in terms of chemotherapy-related toxicities and surgical morbidity; however, the effect did not achieve the expected value. The association between the pRR and survival will be evaluated in the final analysis of this clinical trial.

## Funding

This research received no specific grant from any funding agency in the public, commercial or not-for-profit sectors.

## Conflict of Interest Statement

None declared.
